# Gender aspects in atrial volumetry

**DOI:** 10.1186/1532-429X-13-S1-P222

**Published:** 2011-02-02

**Authors:** Samir Sarikouch, Titus Kuehne, Matthias Gutberlet, Philipp Beerbaum

**Affiliations:** 1 Hannover Medical School, Hannover, Germany; 2 Unit of Cardiovascular Imaging – Congenital Heart Diseases, Deutsches Herzzentrum Berlin, Berlin, Germany; 3 Heart Centre Leipzig, Department of Radiology, Leipzig, Germany; 4 Division of Imaging Sciences, King’s College London, Guy’s & St Thomas’ Hospital, London, UK

## Introduction

The role of atrial size and function is increasingly addressed in acquired and congenital heart disease. Interpretation and further evaluation of atrial parameters are hampered by lack of reference data in growing subjects.

## Methods

We prospectively enrolled healthy 115 children and adolescents, mean age 12.4±4.1 years, range 4.4-20.3, with no acquired or congenital heart disease, no chronic illness or any competitive sports activities. Transversal routine 2D steady-state free-precession acquisition was used to cover the whole heart. We determined maximal and minimal volumes of both atria and calculated cyclic volume change (CVC) and emptying fraction (EMF). Reference centile curves were computed using the lambda-mu-sigma (LMS)-method as described by Cole.

## Results

**Figure 1:**
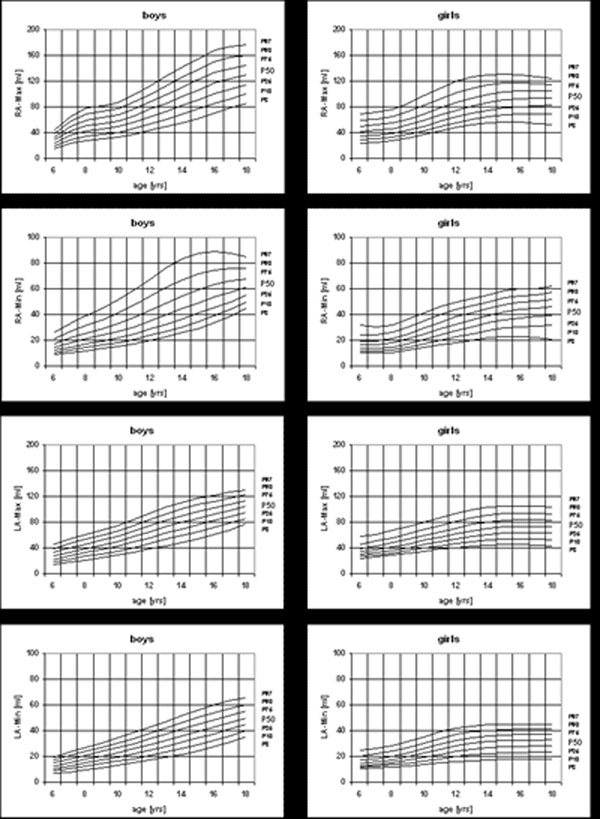
Non-indexed age and gender specific reference percentiles for atrial parameters in children and adolescents from 6-18 years.

Gender differences were noted for atrial volumes and derived parameters. Maximal right atrial volume (RA) for girls was 53.3±11.8 ml/m^2^ and 58.1±15.7 for boys (p=0.064), minimal RA volume for girls/boys was 23.2±6.2/27.0±7.9 ml/m^2^ (p=0.004). Maximal left atrial (LA) volume for girls/boys was 44.2±8.7/46.7±10.1 ml/m^2^ (p=0.143) and minimal LA volume for girls/boys was 19.2±3.9/21.5±5.1 ml/m^2^ (p=0.009). For both atria, cyclic volume change (CVC) was higher for boys, but emptying fraction (EMF) higher for girls (p=0.03). Percentiles of RA/LA volumes showed steeper increase in boys than in girls, who in fact showed a plateau after age 14. Figure 1.

## Conclusions

We established age and gender specific reference percentiles for atrial size and function from 6 to 18 years.

